# Influence of Fuel Moisture Content and Reactor Temperature on the Calorific Value of Syngas Resulted from Gasification of Oil Palm Fronds

**DOI:** 10.1155/2014/121908

**Published:** 2014-01-20

**Authors:** Samson Mekbib Atnaw, Shaharin Anwar Sulaiman, Suzana Yusup

**Affiliations:** ^1^Mechanical Engineering Department, Universiti Teknologi PETRONAS, Bandar Seri Iskandar, 31750 Tronoh, Perak, Malaysia; ^2^Chemical Engineering Department, Universiti Teknologi PETRONAS, Bandar Seri Iskandar, 31750 Tronoh, Perak, Malaysia

## Abstract

Biomass wastes produced from oil palm mills and plantations include empty fruit bunches (EFBs), shells, fibers, trunks, and oil palm fronds (OPF). EFBs and shells are partially utilized as boiler fuel while the rest of the biomass materials like OPF have not been utilized for energy generation. No previous study has been reported on gasification of oil palm fronds (OPF) biomass for the production of fuel gas. In this paper, the effect of moisture content of fuel and reactor temperature on downdraft gasification of OPF was experimentally investigated using a lab scale gasifier of capacity 50 kW. In addition, results obtained from equilibrium model of gasification that was developed for facilitating the prediction of syngas composition are compared with experimental data. Comparison of simulation results for predicting calorific value of syngas with the experimental results showed a satisfactory agreement with a mean error of 0.1 MJ/Nm^3^. For a biomass moisture content of 29%, the resulting calorific value for the syngas was found to be only 2.63 MJ/Nm^3^, as compared to nearly double (4.95 MJ/Nm^3^) for biomass moisture content of 22%. A calorific value as high as 5.57 MJ/Nm^3^ was recorded for higher oxidation zone temperature values.

## 1. Introduction

A tremendous amount of sustainable and renewable raw material for energy use exists in Malaysia in the form of palm oil biomass waste. Oil palm plantation in Malaysia covered 4.98 million hectares of plantation area as of 2011 [1] with estimated harvestable biomass of 50 to 70 tons per hectare per year [2]. Considering the entire life cycle of a palm tree, only 10% of the total plantation is converted to the final product (namely, palm oil and palm kernel oil), while the remaining 90% will be turned to biomass waste [3]. Hence, being a major palm oil exporting country, Malaysia is producing a huge amount of oil palm biomass waste which can be used as an ample and sustainable resource for renewable energy generation [4, 5].

The rise in prices of fossil fuels, their finite and non-renewable existence, and the need for energy independence created a prominent need for renewable and alternative energy sources. In addition, the present extensively increasing use of fossil fuels and the subsequent high rate of pollutant emission is having adverse effects on the environment [6]. Due to these reasons there is a need for adoption of renewable and environmentally friendly energy sources, which are sustainable, less costly, and not contributing to environmental problems [6–8]. One form of renewable and environmentally friendly source of energy is biomass fuel. As Malaysia has an ample source of biomass waste generated from the palm oil industry, it could be utilized as a sustainable, cheaper-and pollution-free alternative energy source.

The biomass waste from the oil palm industry includes empty fruit bunches (EFB), kernel shells, palm oil mill effluent (POME), trunks, and oil palm fronds (OPF). More than 53 million tons of oil palm biomass waste was reported to be produced in Malaysia as of 2009 out of which OPF were found to contribute 24% of the total annual production [4]. To ensure good production rate of the fruit brunch, the fronds (OPF) must be pruned regularly. OPF are available all year round as pruning is done during collection of fresh brunches at the average of two fronds per tree per month. While some of the oil palm wastes like shells and EFB's are partially utilized as boiler fuel, the trimmed OPF are usually left inside the plantations for a low value application of soil nutrition and erosion control. Though there are a number of studies on thermochemical conversion of various biomass fuels, very few numbers of studies exist on oil palm biomass materials. Even the few studies carried out on oil palm biomass are limited to pyrolysis and liquefaction of EFB's, shells, and fibers [3, 9–15]. Only a very recently limited number of studies were reported on fixed bed and fluidized bed gasification of oil palm biomass [16–19], while the studies and recorded data on gasification or pyrolysis of OPF biomass are very few. Hence, the current study is focused on OPF.

Comparison of lignocellulosic composition of OPF with other biomass shows its high potential as a fuel for thermochemical energy conversion. Biomass materials are basically composed of the chemical components cellulose, hemicelluloses, lignin, and ash. The relative composition of these chemical components varies depending on the type of biomass, usually in the range of 40–60% cellulose, 20–40% hemicelluloses, and 10–25% lignin on dry basis [3]. The chemical composition in weight percent of the different oil-palm biomass types, reported in the work of Mohammed et al. [4] and Kelly-Yong et al. [20], were compared with that of OPF. The studies showed that OPF have higher cellulose composition and the least lignin content as compared to other oil palm biomass materials. A study on the effect of woody biomass components on air-water gasification carried out by Hanaoka et al. [21] verified that cellulose has higher carbon conversion efficiency. In addition, higher carbon monoxide composition in the range of 35.5% in syngas was reported compared to hemicellulose and lignin. Moreover, lignin was reported to have inferior qualities in gasification conversion as it mainly consists of fixed carbon compounds. Hence, the higher cellulose composition of OPF (49.8%) and its lower lignin and ash composition (20.5% and 2.4% resp.) indicated that OPF have a higher potential as a gasification feedstock.

The current study focused on the study of downdraft gasification of OPF. Studies on biomass gasification have shown that the major operating parameters that influence the gasification process include air fuel ratio, moisture content of fuel, reactor temperature, and particle size of feedstock [22–26]. This paper studies the influence of moisture content and reactor temperature on the composition and calorific value of syngas, making use of a lab scale downdraft gasification setup. An equilibrium model of downdraft gasification of OPF was developed in order to facilitate prediction of syngas composition and calorific value. Moreover, simulation results from the equilibrium model were compared with experimental data. The detail physical and chemical characterizations and feasibility study of OPF feedstock and the full account of the development of the equilibrium model of gasification were discussed in previous papers [27–35].

## 2. Methodology

### 2.1. Experimental Setup and Procedures

**Figure 1:**
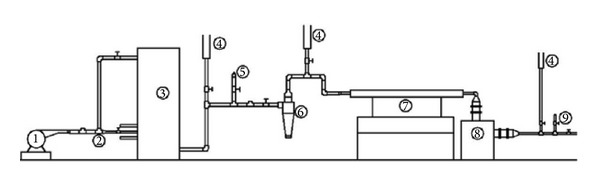
Schematic of the experimental setup: (1) air blower, (2) air distribution line, (3) downdraft gasifier, (4) gas flare points, (5) raw gas sampling point, (6) cyclone for gas cleaning, (7) cooling heat exchanger, (8) oil bath filter, and (9) clean gas sampling point.

The experimental rig used was a batch feed downdraft gasifier. The operation was carried out at atmospheric condition using air as a gasification medium. The design capacity of the gasifier used was 50 kW of thermal output. The reactor was cylindrical with a height of 1000 mm and diameter of 400 mm. A necking or throat of slope angle of 70° was provided near the grate of the gasifier in order to ensure smooth downflow of the biomass by gravity. In order to decrease energy loss due to heat transfer, the gasifier wall was insulated with refractory cement material of thickness 25 mm. The details on the gasifier design were presented in the work of Moni and Sulaiman [36]. The schematic of the experimental setup is shown in Figure 1. The experimental setup consisted of a blower (1) for the supply of air and a number of syngas conditioning units downstream of the gasifier. The conditioning units used for the cooling and cleaning of syngas include a cyclone (6), condenser (7), and oil bath filter (8) which were provided before the cleaned gas sampling point (9). In addition, as shown in Figure 1 a number of flare points (4) were provided on the outlet piping in order to check the combustibility of produced syngas and to burn poisonous gases like CO produced from gasification, before being released to the atmosphere. A multipurpose Emerson X2GP gas analyzer was used to measure volume percentage of the major component gases: CO, CO_2_, CH_4_, and H_2_ in the syngas produced.

The experimental procedure involved collection and recording of temperature readings along the gasifier bed using five type-N thermocouples of accuracy ±2.5°C. The thermocouples were mounted in the middle section at five different points along the length of the gasifier. The temperature readings were collected using USB based temperature data logger at each minute and the readings were automatically stored in a computer. The flow rate of inlet air was measured making use of a VFC Series Dwyer rotameter, which has an accuracy of ±10 lpm. The particle sizes of the feedstock used were between 10 and 25 mm. As the fixed bed gasifier is a batch fed type, 12 kg of OPF was filled in the reactor for experimental test operation. An online gas analyzer unit was used to measure the composition of syngas at the outlet pipe. Besides, the amount of ash, char, and liquid tar produced was collected and weighed at the end of each run, to account for the total material balance of the system and in order to determine the mass conversion efficiency of the gasification process. Once combustion started at the grate, the air inlet blower was connected and started supplying air at a constant flowrate. The gas compositions were recorded every five minutes during the operation. The average of these gas composition values recorded over the steady operation period was used to calculate the calorific value of syngas. The influence of reactor temperature was studied by recording composition of produced syngas corresponding to values of oxidation zone temperature. While the effect of moisture content of fuel on the reactor temperature was studied by carrying out gasification with fuels of different moisture content, the air flow rate was kept constant at 180 lpm for all experiments.

### 2.2. Model Development

Equilibrium model of gasification was developed based on ultimate and proximate properties of OPF considering the batch fed operation which is commonly used in fixed bed gasifiers. The use of equilibrium model is reasonably justified as the batch process allows sufficiently longer time for all the major gasification reactions to reach equilibrium. Moreover, the equilibrium model is known to give sufficiently accurate prediction for processes like gasification that took place at temperature higher than 800°C [37]. The equilibrium model for downdraft gasification of OPF was modeled using the unit operation models of ASPEN Plus process simulation software [30]. Advanced System for Process Engineering (ASPEN) is a software tool that can be used for modeling and simulation of various reaction engineering processes. It is a helpful tool in designing and sizing of reactors as well as predicting chemical process outputs. ASPEN was selected as it handles chemical reactions involving solids and allows the use of external FORTRAN based programing code to enhance the modeling capability. The simulation results from the model were used to predict the composition and resulting heating value of syngas.

The gaseous components considered in modeling of the gasification process include H_2_, O_2_, CO, CO_2_, H_2_O, N_2_, CH_4_, and S. From the different stream classes in ASPEN, the conventional stream class was used for the gaseous reactants and products, while the char produced from gasification was defined as pure carbon. This assumption was adequately valid as the char is known to be composed mainly of carbon. Therefore, conventional solid stream class was used for char. The OPF feedstock and ash were defined as a nonconventional solid making use of the component attribute feature based on ultimate and proximate analysis results of the biomass. The ultimate and proximate analysis results of OPF used as input to the model are shown in Table 1 [2]. Four different unit operation blocks, namely, stoichiometric reactor, yield reactor, and two Gibbs equilibrium reactors were used to model the drying zone, pyrolysis zone, and oxidation and reductions zones, respectively. Full account of the model development and more details on the simulation results were discussed in previous publications [27, 30].

## 3. Results and Discussions

Experiments on downdraft gasification of OPF feedstock with different initial moisture content values were performed. Generally, all the conditions tested resulted in successful gasification operations, of which syngas at the flare point could be ignited. A stable flare was observed for OPF feedstock with lower moisture content (22%), implying that stable gasification temperature was achieved throughout the operation time. Reactor temperature profiles and syngas compositions for gasification experiments with different initial moisture content values of feedstock are compared. In addition, results of the equilibrium model used for prediction of composition and calorific value of syngas at different values of operating parameters for downdraft gasification of OPF are compared with the experimental results.

### 3.1. Typical Gasification Run Results

Average values of the measured syngas composition were used to calculate the calorific value (CV) and gasification efficiency. Summary of the operating parameters and experimental results for the gasification of OPF with moisture content of 22% is shown in Table 2. During the first 30 minutes of the experiment the oxidation zone temperature increased from 100°C to above 800°C. A stable oxidation zone temperature was observed during the subsequent 40 minutes with an oxidation zone temperature values higher than 900°C, implying a steady gasification operation. The high oxidation zone temperature, which was comparable to that for gasification (downdraft) of other biomass fuels, ensured breaking down of heavy hydrocarbon compounds like tar as the gas passed through the oxidation zone [23, 25, 38]. The average calorific value for the dried OPF was 4.95 MJ/Nm^3^ with a peak calorific value of 5.57 MJ/Nm^3^ obtained for oxidation zone temperatures above 1000°C. The average calorific value was found to be comparable with those reported in the literature [23, 25, 39, 40], for other biomass types. Shown in Table 3 is comparison of the calorific values of other biomass materials, which are comparable to OPF. As shown in Table 3, the heating value of syngas obtained from gasification of OPF was comparable with coconut shells and woody biomass materials and higher than hazelnut shells. For downdraft gasification of OPF with initial moisture content of 22%, a gasification efficiency of 70.5% and a mass conversion efficiency of 93.9% were obtained. These efficiency values were found to be within the range of those reported in the literature for downdraft gasification of woody biomass, that is, gasification efficiency of 47 to 88.6% [25, 39] and mass conversion efficiency of 75 to 98% [25, 41]. Hence, the results showed that OPF have a good potential to be used as a gasification fuel and could produce syngas with acceptable heating value. In addition, the gasification and mass conversion efficiencies are comparable to that of woody biomass.

Summary of the typical ASPEN simulation results and the experimental results is shown in Table 4. Both simulation and experimental results were obtained for operating conditions of air fuel ratio (AFR) of 2.07 and initial moisture content of 22%. The comparison of typical simulation results with experimental results was obtained by using the sum square deviation method [42]:

(1) RSS=∑i=1N(yie−yipyie)2,

where *y*_*ie*_ is experimental composition value of each syngas component measured from experiment and *y*_*ip*_ stands for predicted composition of each component from the simulation work. The average mean square error was calculated considering the total number of data, *N*:

(2) MRSS=RSSN.  

Calculation for the mean error was obtained by square root of (2).

The mean errors from the statistical analysis results for comparison between the simulation and the experimental results are shown in Table 4. From the calculated mean errors it can be implied that the predictions of syngas composition from the equilibrium model are generally in good agreement with those from the experimental results. As shown in Table 4 slightly high mean error between predicted and experimental values was obtained for CH_4_ concentration. This is due to the prediction of only trace amount of CH_4_ concentration, from the equilibrium model, as compared to concentrations of 1 to 2.5% obtained in actual gasification. Similar observations were reported in other equilibrium modeling studies [24, 37]. In the work of Gautam [37], CH_4_ concentration lower than 0.15% was predicted by equilibrium model, as compared to 3 to 4% concentration in actual gasification experiments. The prediction from the equilibrium model of gasification in the current study was shown in Table 4 to slightly overestimate the concentration of H_2_ in syngas as compared to experimental results. Similar variations between equilibrium model predictions and experimental concentrations of H_2_ were reported in literature [24, 37, 43]. The heating value of syngas predicted by the equilibrium model was in good agreement with results from actual gasification. The mean error for the prediction of lower heating value (LHV) of syngas was found to be 0.1 MJ/Nm^3^.

### 3.2. Effect of Moisture Content on Reactor Temperature

**Figure 2:**
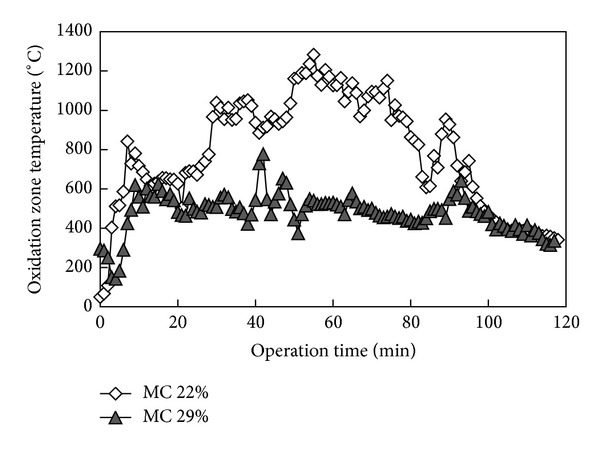
Dynamic temperature profiles of oxidation zone temperature for gasification of fuel with different initial moisture content.

Shown in Figure 2 is variation of oxidation zone temperature with time from the start of batch operation for gasification of fuel with moisture content of 22% and 29%. The results showed that the oxidation zone temperature in the case of the higher moisture content was consistently low during the entire operation duration, with roughly half the average value recorded for lower moisture content. For both cases the oxidation zone temperature showed a sharp rise in the first 10 minutes of operation after startup. The oxidation temperature continued to increase steadily with operation time for the case of lower moisture content fuel, while it remained relatively stable at an average of about 600°C for the case of higher moisture content fuel. Typical for fixed bed gasification process, variations in the temperatures were shown during the operation time. During the relatively stable operation period between the 30th to 80th minutes the average temperature was 1048°C (for low moisture OPF). The reason for fluctuations in temperature could be due to the complex interactions between the number of endothermic and exothermic reactions taking place inside the reactor, as well as because of variations in the direct exposure of the thermocouples to the red hot fuel particles depending on the variations in flow characteristics of the fuel with time [25]. The oxidation zone temperature showed a sharp decrease after the 80th minute of operation due to depletion of the feed material since the gasifier was under batch operation. In addition, flaring of the syngas produced was hardly been possible during the experiment for the case of higher moisture content fuel implying that the high oxidation zone temperature required for various endothermic gasification reactions was not achieved.

### 3.3. Effect of Moisture Content on Gas Composition

**Figure 3:**
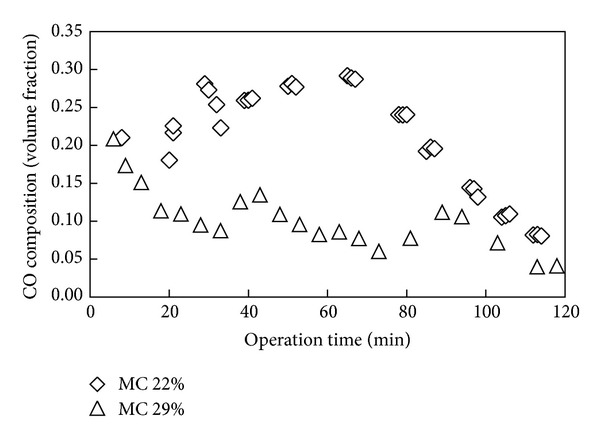
Carbon monoxide content in syngas over operation time for fuel of different initial moisture content.

The effect of initial moisture content of fuel on the composition of syngas throughout the gasification period was studied.  Figure 3 shows the variation of CO content with time from the start of gasification of OPF for different moisture content. It is shown in Figure 3 that for gasification experiment with 22% moisture content of fuel the CO content increases steadily during the initial phase of the gasification but decreases sharply after around the 75th minute of operation. The result showed that the CO content for OPF with 29% moisture content was consistently lower than that of the lower moisture content. An earlier study suggested that CO content in syngas would be favored by increase in the reactor temperature [16], and hence explains the reason for the observation in Figure 3, that is, low CO content for biomass with high moisture content. The sharp decrease in CO composition after the 75th minute of operation could most probably be due to the depletion of the feedstock as the gasifier was batch operated. For gasification of fuel with moisture content of 22%, an average CO content of 25% was obtained during the first 75 minutes of operation time as compared to only 11.4% for the case of high moisture content fuel.

**Figure 4:**
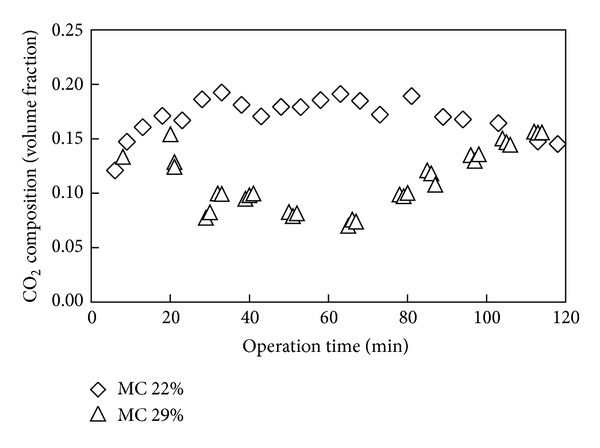
Carbon dioxide content in syngas over operation time for fuel of different initial moisture content.

**Figure 5:**
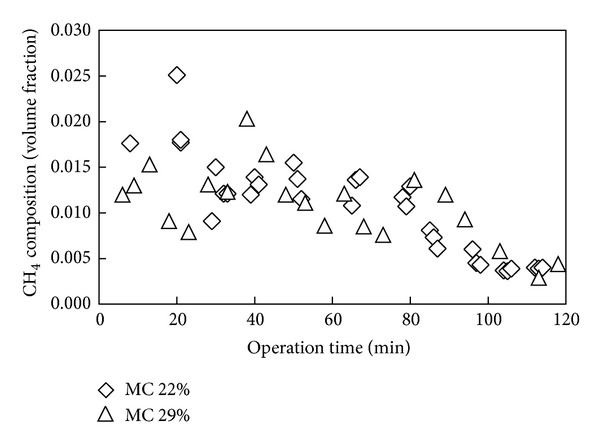
Methane content in syngas over operation time for fuel of different moisture content.

**Figure 6:**
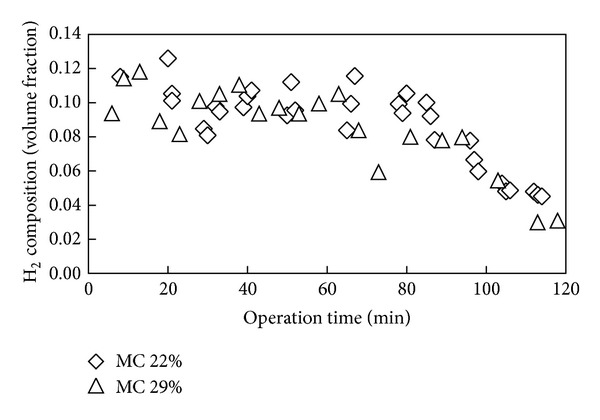
Hydrogen content in syngas over operation time for fuel of different moisture content.

Shown in Figure 4 is variation of CO_2_ content in syngas with operation time for gasification of fuel with moisture content of 22 and 29%. The concentration of CO_2_ is shown in Figure 4 to be lower for higher moisture content value of fuel, indicating that less combustion took place for higher moisture content fuel as evidenced from the lower temperature recorded. The effect of initial moisture content of fuel on CH_4_ and H_2_ composition was only marginal as shown in Figures 5 and 6. Only slightly higher contents of CH_4_ and H_2_ were observed for the case of lower moisture content of fuel, while relatively a similar amount of volume concentration of the gases were found for both fuels. The study of Sulaiman et al. [34] on the effect of fuel moisture content reported that the concentration of H_2_ and CH_4_ increases slightly for gasification of higher moisture content fuel due to steam gasification. Considering the reduction zone reactions [34, 35]:

(3) C+H2O⟶CO+H2   (ΔH=119 kJ/mol)

(4) C+2H2O⟶CO2+2H2 (ΔH=75 kJ/mol)

(5) C+2H2⟶CH4 (ΔH=−87 kJ/mol)

the gasification process is expected to produce higher yield of H_2_, CH_4_, and CO_2_ for the case of higher moisture content fuel, due to steam gasification. However, as the steam-carbon reduction reactions in (3) and (4) are endothermic in nature, the yield of product gases H_2_, CH_4_, and CO_2_ is dependent on the reactor temperature in addition to the moisture content (H_2_O) [34, 35]. Furthermore, part of the H_2_ and CH_4_ in syngas is expected to be produced by the thermal cracking of heavy hydrocarbon compounds like tar. This thermal cracking of heavier hydrocarbons is also possible only at higher temperature. However, during gasification of higher moisture content fuel the oxidation zone temperature was found to be very low, which may be due to the amount of heat consumed during evaporation of the moisture in fuel inside the drying zone. This could be the reason for the production of nearly similar concentration of H_2_ and CH_4_ in syngas for gasification of both high and low moisture content fuel. Hence, it can be concluded from the results that the gasification reaction is more influenced by the reduction in the reactor temperature for high moisture fuel, rather than the effect of steam gasification. Sulaiman et al. [34] also reported that improvement in H_2_ yield from steam-carbon reactions is significant only at higher temperatures above 750°C.

### 3.4. Effect of Moisture Content on Calorific Value of Syngas

**Figure 7:**
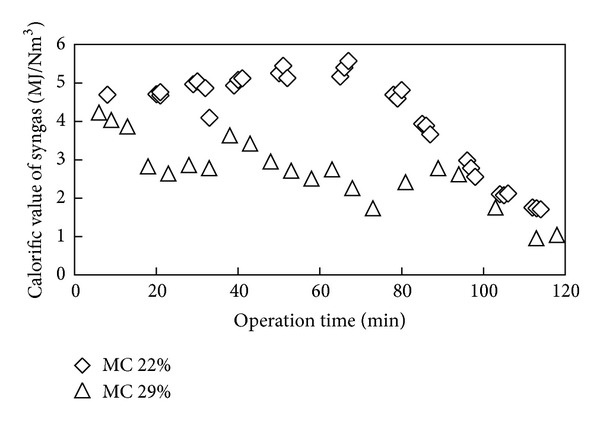
Variation of calorific value with operation time for fuel of different initial moisture content.

Shown in Figure 7 is the variation of calorific value of syngas with operation time for gasification of OPF with moisture contents of 22 and 29%. As shown in Figure 7 consistently low calorific value was observed for gasification of fuel with higher moisture content. This is expected from the lower concentration of CO obtained during the experimental run using high moisture content OPF. The range of calorific values obtained for the case of higher moisture content was below 4 MJ/Nm^3^, which was lower than the expected range of heating value (4–6 MJ/Nm^3^) for syngas produced from typical gasification of woody biomass [25]. On the other hand, the average and peak lower heating values for the gasification experiment with lower moisture content were 4.95 and 5.57 MJ/Nm^3^, respectively. This indicates that initial drying of the feedstock to a lower moisture content value is necessary in order to produce syngas of acceptable heating value. Sulaiman et al. [34] also recommended the use of biomass having low moisture content in order to maximize the heating value and quality of produced syngas. In addition low reactor temperature resulting from high moisture content fuel highly compromises the gas yield, carbon conversion efficiency and gasification efficiency [34]. The drying of the feedstock could simply be done under the sun with no additional energy expenditure and without incurring further cost during the moisture removal. In case of using artificial drying, the additional energy expenditure could be minimized by recovering heat from various streams (such as hot furnace, flu gas from engine or gas turbine, warm air from condenser, etc.) in a commercial gasification power plant [36]. Hence, further studies need to be done in future in order to reduce the impact of energy expenditure, for pretreatment of the feed in terms of drying, on the overall efficiency of the gasification plant.

### 3.5. Effect of Reactor Temperature on Syngas Calorific Value

**Figure 8:**
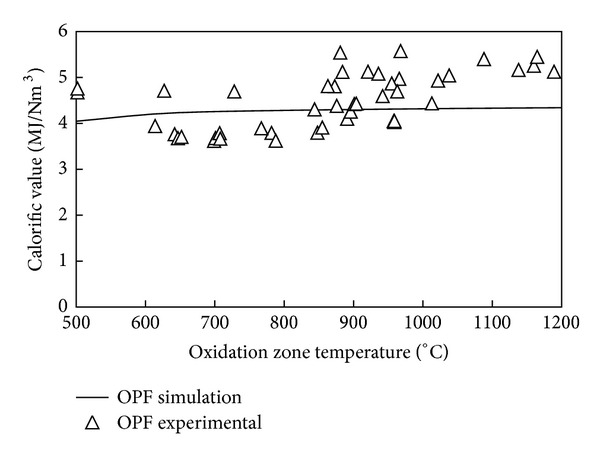
Variation of predicted and measured calorific value of syngas with oxidation zone temperature.

The calorific value (CV) of the producer gas is highly important for consumption as fuel in engines for power generation. As the main objective is to obtain a high calorific value fuel gas with consistent energy density, the effect of reactor temperature on the calorific value of producer gas was investigated. The experimental study on variation of syngas calorific value with temperature was carried out by recording the composition of produced syngas corresponding to different values of reactor temperature. For the modeling study, initially syngas composition was predicted using the equilibrium model of gasification. Then the lower heating value (LHV) of producer gas from OPF gasification was estimated by using the predicted fractions of gases, *X*_*i*_, and heating value of the major fuel components CO, H_2_, and CH_4_ making use of the relation:

(6) LHVsyngas  =XCO×(LHV)CO+XH2 ×(LHV)H2+XCH4×(LHV)CH4,

where the LHV of the major fuel components: CO, H_2_ and CH_4_ were taken to be 13.1 MJ/Nm^3^, 11.2 MJ/Nm^3^, and 37.1 MJ/Nm^3^, respectively [40, 44]. Shown in Figure 8 is the variation of predicted and experimental values of calorific value of syngas over the range of oxidation zone temperature between 500 and 1200°C. Both the equilibrium model and experimental results showed an increase in heating value of syngas with increase in temperature as shown in Figure 8. A similar trend of increase in heating value of syngas with reactor temperature was obtained in the work of Lahijani and Zainal [16] for fluidized bed gasification of EFBs and sawdust biomass. The experimental calorific value results were shown in Figure 8 to be slightly higher than the predicted values for temperatures above 850°C. This could be explained in terms of the increase in concentration of CO in syngas with operation time. At the start of the gasification operation the reactor temperature would be low and it started to increase steadily with time. The temperature exceeded 850°C after about 30 minutes of operation. Due to the batch fed nature of the gasifier, most of the fuel's hydrogen based volatile contents underwent devolatilization, and what remained at later operation time (when the reactor temperature was higher than 850°C) was mostly char. This contributes to the high rate of CO production at higher reactor temperature and this was not captured in the ASPEN PLUS simulation. As CO was the main fuel component with significant amount of heating value in syngas, its higher concentration resulted in a slightly higher calorific value of syngas for the experimental results as compared to model predictions at higher temperature. However, the experimental and model prediction results showed satisfactory agreement with a mean error of only 0.017 MJ/Nm^3^ for the range of oxidation zone temperature of between 500 and 1200°C.

## 4. Conclusions 

Experimental investigation on effect of moisture content of fuel and temperature on syngas composition and calorific value was carried out. Lower composition of carbon monoxide was observed for higher moisture content fuel. The effect of moisture content on H_2_ and CH_4_ concentrations was only marginal with slightly higher values recorded for gasification of low moisture content fuel. For a biomass moisture content of 29%, the resulting calorific value for the syngas was found to be only 2.63 MJ/Nm^3^, as compared to nearly double (4.95 MJ/Nm^3^) for biomass moisture content of 22%. In addition, all data points recorded for gasification of acceptable moisture content fuel were within the range of 4–6 MJ/Nm^3^, as expected for atmospheric gasification of biomass. This indicates that initial drying of the feedstock to lower moisture content value is necessary in order to produce syngas of acceptable heating value and it has a huge advantage in terms of the production of a high calorific value. In addition, predictions from equilibrium model of downdraft gasification of OPF, developed for the prediction of syngas composition and calorific value, were shown to be in good agreement with the experimental results. The experimental and model prediction results when compared over the range of oxidation zone temperature of between 500 and 1200°C showed satisfactory agreement with a mean error of only 0.017 MJ/Nm^3^. Both experimental and simulation results ascertain that calorific value increases with reactor temperature. Calorific value as high as 5.57 MJ/Nm^3^ was recorded at oxidation zone temperature of above 1000°C. The results of the study demonstrated that drying of the OPF feedstock to low moisture content and maintaining a high oxidation zone temperature could significantly improve the gasification output.

## Figures and Tables

**Table 1:** Ultimate and proximate analysis of OPF [2].

Proximate analysis (%)*	
Volatile matter (VM)	85.1
Fixed carbon (FC)	11.5
Ash	3.4

Ultimate analysis (%)*	
C	42.4
H	5.8
N	3.6
O (by diff.)	48.2

*Dry weight basis.

**Table 2:** Operation parameters and results for a typical gasification test operation.

Type of feedstock	OPF
Total operation time (min)	118
Moisture content (% wet basis)	22
Initial weight of feed (kg)	12
Inlet air flow rate (lpm)	180
Air fuel ratio (kg air/kg fuel)	2.07
Equivalence ratio (ER)	0.44
Average oxidation zone temperature (°C)	778
Average reduction zone temperature (°C)	510
Average pyrolysis zone temperature (°C)	456
Average drying zone temperature (°C)	222
Estimated gas yield (lpm)	281.2
Average calorific value of syngas (MJ/Nm^3^)	4.95
Weight of ash and char (g)	620
Amount of tar produced (g)	107.8
Gasification efficiency (%)	70.54
Mass conversion efficiency (%)	93.94

**Table 3:** Comparison of heating value of syngas from gasification of different biomass.

Biomass type	LHV (MJ/Nm^3^)	References
OPF	4.95	Current study
Coconut shells	4.9	[40]
Hazelnut shells	4.70	[23]
Furniture wood	5.62	[25]
Woody biomass	5.02	[24]

**Table 4:** Comparison of typical simulation results with experimental values.

Components	Typical	Experimental results at different operation time (min)								
	simulation	**10**	20	30	40	50	60	70	80	Mean*
	results									error
CO	19.47	21.02	18.07	28.12	25.93	26.22	27.70	28.89	24.01	0.21
CO_2_	13.99	13.34	15.43	7.73	9.48	9.97	8.15	7.58	9.72	0.47
H_2_	15.57	11.51	12.59	8.47	9.73	10.71	9.54	9.93	9.39	0.49
CH_4_	0.01	1.76	2.51	0.91	1.20	1.31	1.15	1.36	1.07	0.86
N_2_	54.38	52.37	51.40	54.77	53.66	51.79	53.46	52.24	55.81	0.04
LHV (MJ/Nm^3^)**	4.30	4.70	4.71	4.97	4.93	5.12	5.12	5.40	4.59	0.10

*Mean error between typical simulation results and experimental data based on sum square deviation method.

**LHV: low heating value.
